# A new palliative surgical technique for high risk Total anomalous pulmonary venous connection (Sarmast-Takriti shunt)

**DOI:** 10.1186/s13019-019-0953-4

**Published:** 2019-07-01

**Authors:** Hossein Sarmast, Ahmad Takriti

**Affiliations:** Department of Cardiac Surgery Hospital, Damascus university, Mouasat Square Omar ben Abdulaziz Street, Damascus, Syria

**Keywords:** Total anomalous pulmonary venous connection - Sarmast, Takriti shunt (STS), Pulmonary venous obstruction, Low birth weight- pulmonary venous confluence -palliative surgical technique- congenital heart disease-cardiopulmonary bypass

## Abstract

**Background:**

Total Anomalous Pulmonary Venous Connection (TAPVC) is a rare heterogeneous condition That accounting for 1.5–3% of congenital heart diseases. It is characterized by failure of the Pulmonary Venous Confluence (PVC) to be directly connected to the left atrium in combination with a persistent splanchnic connection to the systemic venous circulation. The most critical status occurs when it is accompanied by pulmonary venous obstruction. Managing of this situation is very difficult and in fact, pulmonary venous obstruction is usually lethal. The real aim of this study is offering a new palliative surgical technique (Sarmast – Takriti Shunt) in order to alleviate the patient’s signs and symptoms until becomes ready for the main surgical correction.

**Case presentation:**

The study included a 4–day old, low birth weight boy who suffered from Critical Obstructive Total Anomalous Pulmonary Venous Connection. The decision was made to perform the new palliative technique using Gore - Tex (ePTFE). Anastomosis was established without Cardiopulmonary Bypass (CPB) between Pulmonary Venous Confluence (PVC) and the left atrial appendage. Therefore the Sarmast – Takriti Shunt (STS) was taken place.

**Conclusion:**

After completion of the procedure, the pressure gradient across the venous confluence and the Left innominate vein became zero. Cyanosis, agitation and feeding Problem subsided. Three days later, when he was discharged, arterial oxygen saturation had reached as high as 91%. After 7 months we perfomed the main correction.

## Background

Total anomalous pulmonary venous connection is a rare heterogeneous anomaly,accounts for 1.5–3% of congenital heart diseases [1]. It is characterized by abnormalreturn of whole Pulmonary venous blood flow to the right atrium or systemic venous tributaries due to its persistent splanchnic connection [2]. A concomitant right to left shunt, commonly via an interatrial communication, is required for survival after birth. Darling classified it in four categories: Supra-cardiac 45%, cardiac 25%, Infra-cardiac 25% and mixed type 5–10% [3]. At one end of the spectrum, there are completely unobstructed circulation, these neonates present with a large left to right shunt manifestations. At the other end there are severe PVO neonates born with TAPVC have poor prognosis with approximately 80% mortality in the first year of life. Both obstructed and non-obstructed types of TAPVC pose an absolute indication for surgical repair [4]. In PVO type without intervention the median survival is 2 months, with the shortest survival being 1 day. Despite greatly improved neonatal care and surgical techniques over the last decade, TAPVC operation is still associated with high hospital mortality, up to 20% [5, 6].

## Case presentation

**Fig. 1:**
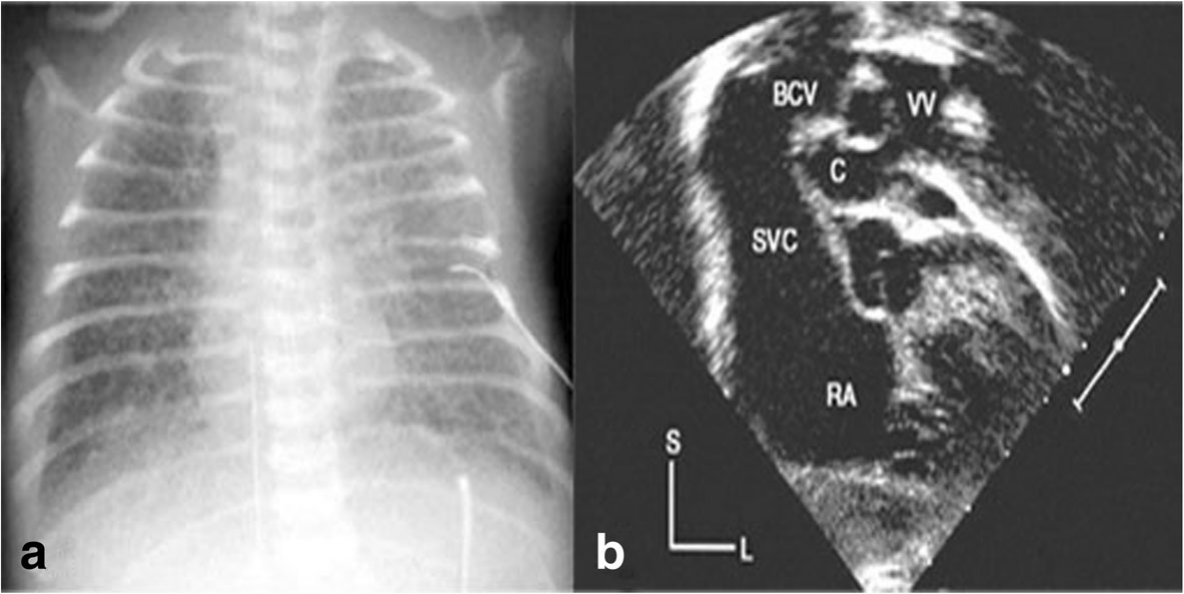
4-days old male with total anomalous pulmonary venous connection accompanied by pulmonary venous obstruction: a- CXR: Note mild cardiac enlargement and evidence of pulmonary venous hypertension (“ground glass” appearance). b- 2D-Echocardiography shows compressed vertical vein between lt.pulmonary artery and lt.main.bronchus

**Fig. 2:**
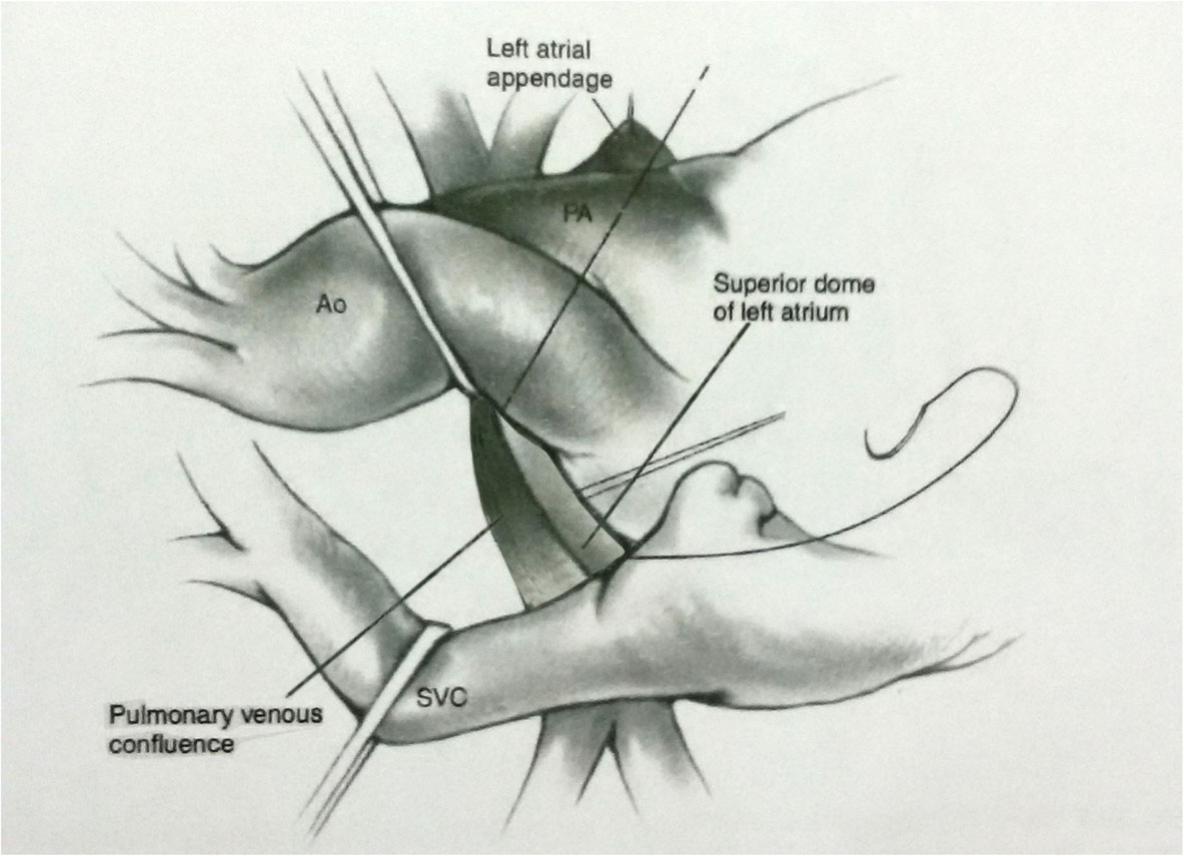
Schematic illustration of superior approach in 4-days old male with total anomalous pulmonary venous connection

**Fig. 3:**
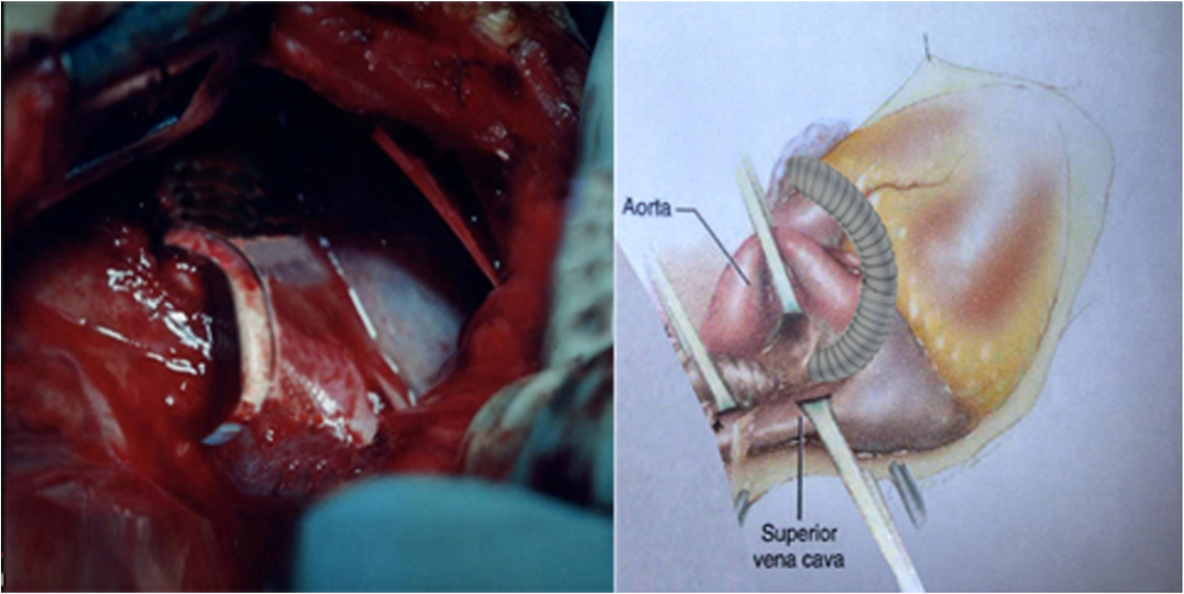
Schematic and operational photo illustrations of sarmast - takriti shunt (STS) in 4-days old male with total anomalous pulmonary venous connection

A 4 –day old, low birth weight boy (w = 1950 g) was presented to our departmentwith discrete but increasing cyanosis, tachypnea, respiratory distress, hepatomegaly, hypoxia (Sao2 = 70%), gasping, poor feeding and severe metabolic acidosis. The prenatal course was uneventful and he was born by normal vaginal delivery on gestational age = 38.5 w. The patient didn’t carry any congenital heart disease (CHD) history in his genetically close relatives (first, second and third degree). Immediate and brief work up was carriedout. Chest X Ray (CXR) showed normal heart size with ground glass appearance in all the lung fields (Fig. 1a). Color doppler and 2 D- echocardiography revealed the total anomalous pulmonary venous connection (TAPVC – supra cardiac type), accompanied by significant gradient between the drainage point of vertical vein to the left brachiocephalic vein and the pulmonary veins with flow acceleration > 3.0 m/sec (pulmonary venous obstruction). It was also uncovered presence of the ASD secundum, as the natural last resort for being alive. The vertical vein was noted to be compressed as it coursed posterior the left pulmonary artery and anterior the left main bronchus (Fig. 1b). According to the aforementioned findings, the boy had almost met most of incremental risk factors leading to mortality after conventional operations. Therefore the decision was made to a new palliative surgical procedure for the first time. The patient suffered from critical pulmonary venous obstruction (PVO) with severe hypoxia, metabolic acidosis and Pulmonary hypertension (PHT) besides the restricred time for preoperative evaluation and preparation, therefore in order to preoperative medical stabilizing we conventionally used 100% O2 with the aim of promoting respiratory alkalosis as well as nitric oxide as a pulmonary vascular dilator as a last resort. Under general anesthesia, median sternotomy and partial thymectomy were carried out. The pericardium was opened in vertical fashion then prudent purse-string sutures as stand by were placed on ascending aorta and right atrial appendage (without using CPB). After intravenous heparinization (100 U/ kg), at first some dissections were done from left lateral side between heart and pulmonary venous confluencethen the dome of the left atrium was exposed. The posterior pericardium just superior the dome of LA was incised and PVC was appeared (Fig. 2). Using a side – biting clamp on the PVC, a longitudinal incision was made. The proximal head of a Gore -Tex (ePTFE) with appropriate size (diameter = 6 mm) that had been prepared and beveled, was anastomosed to PVC using continuous 6–0 polypropylene suture. Under topical cooling of heart with aim of more and better protection of heart in case we didn’t use CPB as well as for control of cardiac movements, and using a side-biting clamp on left atrial appendage(LAA), the distal end of Gore-Tex was anastomosed to LAA. After deairing with heparinized saline as routine, the clamp was removed. The Sarmast -Takriti Shunt (STS) between PVC and LA was established (Fig. 3). Immediately after completion of the procedure, cyanosis began to decrease. In order to the prophylactic anticoagulant therapy, we initially administered low dose rectal ASA (5 mg/kg/d) followed by oral route (10 mg/kg/d) from the POD = 1. We performed the main operation 7-months later with excellent outcome when he had already sustained satisfactory weight (w = 7030 g), as follows: After the establishment of CPB, the shunt was removed. To reduce the the risk of residual obstruction of PVC due to pocket-like contraction our team preferred modified septosuperior approach (komarakshi technique). A direct anastomosis between PVC and L. A, ligation of the VV and closure of ASD with autopericardial patch were achieved in one stage repair.

## Discussion and conclusions

Evaluation of pressures before intervention in the operation room and after correction are illustrated in the (Table 1.). Immediately after completion of surgery (STS), the pressure of PVC decreased to the point where its pressure gradient became zero. Blood oxygenation improved up to 84% (preoperative Sao2 was 70% on 100% oxygen) and cyanosis, agitation, feeding problem subsided. Three days later, when he was discharged, arterial oxygen saturation had reached as high as 91%. Despite good advances in treating of TAPVC in recent decades, this severe malformation in its various anatomical forms remains a challengeable entity during early infancy. Significant obstruction to pulmonary venous drainage results in pulmonary edema in the presence of a normal size and shape of the heart and cardiogenic shock which is rapidly lethal if untreated. Our patient preoperatively suffered from significant gradient between the drainage point of vertical vein to the left brachiocephalic vein and the pulmonary veins with flow acceleration > 3.0 m/sec (pulmonary venous obstruction) but in postoperative echocardiography, midflow acceleration 0.8 m/sec was found at the conduit site. This flow and patency was maintained up to the second operation, showing an identical median Vmax of 0.8 m/sec. Neither turbulent nor continious flow was observed in the conduit. Almost all reports have declared that perioperative high mortality associates with PVO, low weight (W < 2.5–3 kg), early age (A < 2 m), severe preoperative acidosis, long time of aortic Cross Clamp (ACC) and cardiac arrest. The second frontier in the treatment of TAPVC is represented by postoperative PVO. In such a difficult situations, if patients survive from operation, most of them will require multiple postoperative surgical interventions due to recurrent PVO with an increasingly poor outcome at each representation [7]. Medical efforts are minimally effective in managing the ensuing hemodynamic and metabolic problems so their use is limited to provide some short lived conservative therapy until definitive surgical treatment is carried out. PVO is usually lethal, even with reoperation and extensive attempts at revision or repair [8]. This lack of success has led to alternative treatments such as balloon dilatation and stenting. The Rashkind Operation or Balloon Atrial Septostomy (BAS) has been used with some success to decompress the pulmonary venous pressure and improve C/O in the restricted ASD, but these don’t appear to provide additional benefit. Moreover several reportshave proposed the use of percutaneous angioplasty and stenting of the obstructed vein to palliate shock and improve preoperative metabolic state. Research showed during the median cross - sectional follow up of 3.1 years, estimated mortality was 38+/− 8% at 1 year and 50+/− 8% at 5-years after stent implantation. Necessity for reintervention (owing to occlusion of stent), was 58+/− 7% at 1-year. In 1996 sutureless repair technique was described, using in situ autologous pericardium for recurrent pulmonary vein stenosis following main TAPVC surgery [9]. Subsequent reports emphasize the utility of this technique in selected patients as main procedure. Despite interest in the sutureless technique, there is little firm evidence that it provides a benefit over conventional techniques used a retrospective analysis to compare the outcomes of death and restenosis after conventional and sutureless techniques. By multivariable analysis, there was no statistically significant difference between the conventional and sutureless techniques. We encountered with a patient, who had almost encompassed all critical risk factors that were sufficient to make the operative prognosis very poor. Routine conventional operative procedures have reported mortality rate up to %50 and post operative pulmonary venous obstruction (PVO) morbidity rate up to %54 especially in the obstructive type of TOPVC as well as almost all of them need to the secondoperation. Our patient took more opportunity to gain appropriate weight also heart chambers and pulmonary veins grew adequately. The Sarmast - Takriti Shunt (STS) stablished adequate postoperative hemodynamics for symptomatic neonate and prompt left cardiac side rehabilitation. The STS with confined heparin (100 U/kg), was carried out without using CPB with an intention to reduce the morbidity associated with extra corporeal circulation. Eliminating CPB reduced the cost of the procedure substantially and saved the patient from its inherent complications [10]. .After procedure the enough time was prepared on behalf of the heart to compensate its chambers especially the right ventricle and left atrium and ensure endurable state for the main surgery. Although our experience was limited to STS in supracardiac type, we are optimistic and hopeful to its feasibility and usefulness in other types of TAPVC. Now, we are so satisfied owing to be able to help such a complicated neonate.

**Table 1:** Preoperation and postoperative cardiac pressures of 4-days old male with total anomalous pulmonary venous connection accompanied by pulmonary venous obstruction

	PVC mean pressure	Left brachiocephalic vein	Left atrium	Right atrium	Right ventricle	Pulmonary artery
Preoperative pressures (mmHg)	29	9	8	9	61/13	59/31
Postoperative pressures (mmHg)	8	8	9	8	32/10	28/15

## Data Availability

The datasets during the current study available from the corresponding author on reasonable request and for this purpose, the authors received written consent from patient’s parents.

## Abbreviations

ACC: Aortic cross Clamp
ASD: Atrial septal Defect
BAS: Balloon atrial Septostomy
C/O: Cardiac output
CHD: Congenital heart Disease
CPB: Cardiopulmonary Bypass
LA: Left atrium
PHT: Pulmonary Hypertension
POD: Post operative Day
PVC: Pulmonary Venous Confluence
PVO: Pulmonary Venous Confluence
STS: Sarmast _Takriti Shunt
TAPVC: Total anomalous Pulmonary Venous Connection
VV: Vertical vein
